# Effect of Non-invasive Vagus Nerve Stimulation on Resting-State Electroencephalography and Laser-Evoked Potentials in Migraine Patients: Mechanistic Insights

**DOI:** 10.3389/fnhum.2018.00366

**Published:** 2018-09-13

**Authors:** Eleonora Vecchio, Iege Bassez, Katia Ricci, Cristina Tassorelli, Eric Liebler, Marina de Tommaso

**Affiliations:** ^1^Applied Neurophysiology and Pain Unit, SMBNOS Department, Polyclinic General Hospital, Bari Aldo Moro University, Bari, Italy; ^2^Department of Data Analysis, Ghent University, Ghent, Belgium; ^3^Headache Science Center, C. Mondino Foundation, Pavia, Italy; ^4^Department of Brain and Behavioral Sciences, University of Pavia, Pavia, Italy; ^5^electroCore LLC, Basking Ridge, NJ, United States

**Keywords:** vagus nerve stimulation, EEG, laser-evoked potentials, migraine, headache

## Abstract

A recent multicenter trial provided Class I evidence that for patients with an episodic migraine, non-invasive vagus nerve stimulation (nVNS) significantly increases the probability of having mild pain or being pain-free 2 h post-stimulation. Here we aimed to investigate the potential effect of nVNS in the modulation of spontaneous and pain related bioelectrical activity in a subgroup of migraine patients enrolled in the PRESTO trial by using resting-state electroencephalography and trigeminal laser-evoked potentials (LEPs). LEPs were recorded for 27 migraine patients who received active or sham nVNS over the cervical vagus nerve. We measured power values for frequencies between 1–100 Hz in a resting-state condition and the latency and amplitude of N1, N2, and P2 components of LEPs in a basal condition during and after active or sham vagus nerve stimulation (T0, T1, T2). The P2 evoked by the right and the left trigeminal branch was smaller during active nVNS. The sham device also attenuated the P2 amplitude evoked by the left trigeminal branch at T1 and T2, but this attenuation did not reach significance. No changes were observed for N1 amplitude, N1, N2, P2 latency, or pain rating. nVNS induced an increase of EEG power in both slow and fast rhythms, but this effect was not significant as compared to the sham device. These findings suggest that nVNS acts on the cortical areas that are responsible for trigeminal pain control and pave the ground for future studies aimed at confirming the possible correlations with clinical outcomes, including the effect on symptoms that are directly correlated with trigeminal pain processing and modulation.

## Introduction

Non-invasive Vagus Nerve Stimulation (nVNS) is a promising new treatment for migraine. The efficacy of cervical nVNS (gammaCore^®^, electroCore, LLC) in primary headache prevention or acute therapy has been reported in multiple trials (Simon and Blake, 2017). A recent multicenter, double-blind, randomized, sham-controlled trial, provided Class I evidence that for patients with an episodic migraine, nVNS significantly increases the probability of having mild pain or being pain-free 2 h post-stimulation (Tassorelli et al., 2018). The mechanism of action of VNS in the treatment of headache is probably multifactorial and can be examined in the possible mechanisms involved in modulation of migraine pain.

Previous work in animal models has shown that VNS activates the nucleus tractus solitarius, locus coeruleus, and dorsal raphe nuclei (Dorr and Debonnel, 2006; Cunningham et al., 2008; Ay et al., 2015). fMRI studies demonstrated the action of vagal nerve activation. The majority of afferent vagal fibers enter the brain through the jugular foramen and synapse onto the nucleus tractus solitarius, the first central relay of vagal afferents, which then project directly and indirectly to various structures in the brain (e.g., locus coeruleus, dorsal raphe nucleus, periaqueductal gray) implicated in the mechanism of action of VNS in epilepsy (Beekwilder and Beems, 2010). Frangos and Komisaruk (2017) compared the effects of nVNS vs. control sternocleidomastoid muscle stimulation and reported that nVNS activated the nucleus tractus solitarius and parabrachial area, primary sensory cortex, basal ganglia, frontal cortex, and insula. Deactivations were found in the hippocampus, visual cortex, and spinal trigeminal nucleus. These changes were similar to those reported in fMRI studies of invasive VNS in patients with epilepsy and in a study from the same group on the effects of nVNS applied to the external ear in regions innervated by the auricular branch of the vagus nerve (Frangos et al., 2015).

Oshinsky et al. (2014) described the effects of nVNS in a rat model of trigeminal allodynia. Collectively, these animal data suggest that nVNS may treat headache pain by modulating afferent fibers to the trigeminal nucleus caudalis and therefore its projections to the thalamus and cortex. nVNS also inhibits cortical areas linked to the CSD phenomenon and was recently shown to rapidly inhibit CSD in rodents, providing important insights into the mode of action of this therapy (Chen et al., 2016).

Based on these animal models and human fMRI evidence, three mechanisms of action may be implicated for the effect of nVNS in treating migraine attacks: a central modulation of the cortical and subcortical sites involved in descending pain modulation, a direct modulation of nociceptive afferents to the trigeminal spinal nucleus, or an inhibiting effect on the bioelectrical activity and possibly on CSD phenomena that lead to trigemino-vascular system activation. This latter hypothesis seems less compelling in the case of migraine attacks without aura, where the existence of the CSD phenomenon is unclear and the resolution of CSD not clinically detectable (Ayata, 2010; Borgdorff, 2018).

Laser-evoked potentials (LEPs) are a validated tool to analyze the status of the nociceptive afferent pathways (Treede et al., 2003). In previous studies, we described that LEPs induced via the stimulation of trigeminal A-delta fibers increased in amplitude during the ictal phase and were attenuated by acute pharmacologic therapies for migraine (de Tommaso et al., 2005b). More recently, we showed that electrical stimulation of cutaneous trigeminal afferents induces specific changes in LEPs induced by painful laser stimuli delivered to the right forehead (Vecchio et al., 2017). In both studies, vertex wave amplitudes decreased with pharmacological and non-pharmacological interventions that have proven clinical efficacy for the acute treatment of migraine attacks (de Tommaso et al., 2005c; Vecchio et al., 2017).

The possible effect on CSD is difficult to be demonstrated. The scalp EEG showed CSD in patients with aneurismal subarachnoid hemorrhage and hemispheric stroke, as well as in patients suffering from brain trauma, while during migraine aura the critical EEG signal is not flattened (Drenckhahn et al., 2012; Borgdorff, 2018). In any case, we included the analysis of nVNS effects on resting state EEG, in the hypothesis that a clear action on the brain rhythms could in some way interfere with the evolution of CSD.

The aim of this study was to use LEPs to investigate the potential effect of nVNS for the modulation of pain in a subgroup of migraine patients enrolled in the PRESTO (ClinicalTrials.gov identifier: NCT02686034). More specifically, we tested the effects of nVNS compared to sham stimulation on:

(1) Resting-state electroencephalography (EEG).
(2) Trigeminal laser-evoked responses.

## Materials and Methods

### Patients

The 40 patients selected at our center for the PRESTO study were requested to participate in this randomized sham-controlled sub-study. Selection criteria for patients in the PRESTO trial have been reported by Tassorelli et al. (2018). Patients with peripheral nerve diseases, diabetes, and other potential causes of peripheral nerve involvement were excluded. The protocol for this neurophysiological study was approved by the Ethical Committee of Bari Policlinico General Hospital. All patients provided their written informed consent in accordance with the Declaration of Helsinki.

### Experimental Procedures

Patients underwent EEG and LEP recordings on the day of randomization to the active or sham device. Both the technical staff who performed the EEG and LEP recordings and the examiners of the EEG and LEPs were blinded.

Non-invasive vagus nerve stimulation was delivered using a portable CE-marked device (gammaCore) placed over the expected location of the vagus nerve in the anterolateral cervical region. This hand-held nVNS device produces a low-voltage electrical signal consisting of a 5-kHz sine wave burst lasting for 1 ms (five sine waves, each lasting 200 ms) repeated once every 40 ms (25 Hz) on each side of the neck, which are the stimulation parameters used for treating migraine attacks (Tassorelli et al., 2018). The sham device produced a low-frequency biphasic signal that could be felt as a variable tingling sensation but was not intended to stimulate the vagus nerve. The nVNS and sham devices were otherwise identical in appearance, weight, visual and audible feedback, and user application (Tassorelli et al., 2018).

**FIGURE 1:**
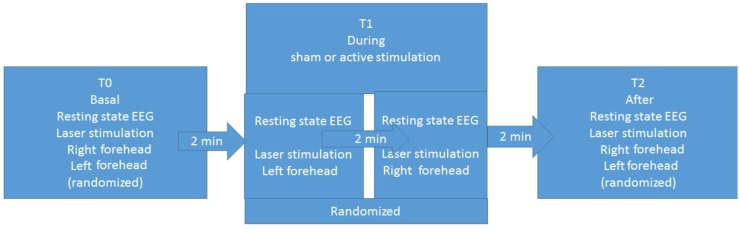
Study design.

The experimental procedure was based on three sessions for each patient: basal T0, during stimulation T1, and after stimulation T2 (**Figure 1**). Each session included resting-state EEG and laser stimulation. Patients were requested to remain quiet with their eyes open, while focusing on a fixed point in the center of a monitor. In the three sessions, we recorded LEPs, while stimulating the right and left forehead, corresponding to the cutaneous territory of the first trigeminal branch. In the T1 session, we recorded LEPs and resting-state EEG during the active or sham stimulation. The order of stimulation sites was randomized across patients (**Figure 1**). The nVNS stimulations were delivered at the maximum stimulation intensity tolerated by the patient (Tassorelli et al., 2018).

### EEG and Laser-Evoked Potential Procedures

The patients laid on a couch in a warm, semi-darkened room. In preliminary recordings we used 32 surface electrodes, then, after the first round of analysis, we decided to increase the number of electrodes, using a 61 channels montage. The recording electrodes were placed on the scalp referred to the nasion, according to the International 10–20 System. The recording system was a MICROMED EEG apparatus (Micromed Brain Quick, Mogliano Veneto, Italy). Two additional electrodes were positioned above the eyebrows for the electrooculogram recording. The impedance was kept below 4 KΩ. During the recording sessions, we applied digital filters in the 0.1–70 Hz range and a notch 50 Hz filter to allow signal inspection.

For the resting-state EEG, we considered the 10 s preceding the laser stimulation, and the inter-stimulus intervals, focusing on the 4 and 5°s following each stimulus. Longer periods were not allowed for the short duration of the active and sham stimulations.

For LEP stimulation, cutaneous heat stimuli were delivered by a CO_2_ laser (wavelength, 10.6 mm; beam diameter, 2 mm; ELEN, Florence, Italy) on the right and left supraorbital zones. The duration of the stimulus was 30 ms. The laser pain threshold (Pth) was established by delivering a series of stimuli at increasing and decreasing intensities using 0.5-W steps. The pain threshold was the lowest intensity that enabled at least 50% of the stimuli to be perceived as a painful pinprick. The laser intensity was two 0.5-W steps over the Pth, in accordance with previous studies (Treede et al., 2003; de Tommaso et al., 2017). We asked all patients to rate the laser pain on a visual analog scale after each trial of stimulation. In the 0–100 visual analog scale, the white color corresponding to *0* indicates no pain sensation and the intense red corresponding to *100* indicates the worst pain conceivable. For each stimulation site and each condition, we delivered one series of 20 laser stimuli, with an inter-series interval of 2 min and an inter-stimulus interval of 10 s. To avoid damage to the skin, fatigue, or sensitization of nociceptors, we shifted the irradiated spot after each stimulus.

### Analysis

We evaluated resting state EEG features in patients recorded with 61 EEG channels (10 patients in sham and 10 patients in real nVNS group).

For resting-state EEG analysis, preprocessing was performed in MATLAB, using the EEGLAB 14_1_1 tool. The pipeline was based on Makoto’s pre-processing pipeline^1^ with some adaptations. The data were first high-passed filtered at 1 Hz to remove slow drifts. Next, a notch filter at 50 Hz (L: 48, H: 52) and 100 Hz (L:99, H: 101) was applied to remove power line noise artifacts. We applied the automatic approach referred to as *Artifact Subspace Reconstruction* to correct continuous data and reject bad channels and data segments. All removed channels were subsequently interpolated and the data were re-referenced to the average. Independent-component analysis was performed (*runica* EEGLAB function, using the *pca* option because of the rank deficiency as a result of interpolating and average referencing). Artifactual components were then automatically removed by using a machine learning algorithm referred to as the *Multiple Artifact Rejection Algorithm* (Winkler et al., 2011). The Multiple Artifact Rejection Algorithm has been shown to perform well in rejecting muscle artifact components (Winkler et al., 2011), which was important in the current study. ICA was performed on the *continuous* data, and artifactual IC were removed using the Multiple Artifact Rejection Algorithm. Lastly, a Laplacian filter was performed on the data. The EEGLAB *spectopo* function was used to calculate the power spectrum. This function uses the pwelch method (*pwelch* function from the MATLAB signal processing toolbox). We used default settings with a window length of 256, fft length of 256, and 0 overlap. We obtained power values for frequencies between 1–100 Hz in steps of 1 Hz. Data points corresponding to 24, 25, 26, 49, 50, 51, 74, 75, 76, and 100 Hz were removed to be certain that no differences would be found that could be attributed to power line noise artifacts or artifacts due to the frequency harmonics of the gammaCore device. This left us with 91 frequency points between 1–99 Hz. We then took pairwise differences between T0 and T1 and between T0 and T2 for all channels. Next, we split the data into frequency bands (delta-theta: 1–7 Hz, alpha: 8–12 Hz, beta: 13–29 Hz, gamma: 30–99 Hz) and averaged the difference power values of the frequencies within a band.

Laser-evoked potential analysis was performed by an investigator who was blinded to the procedure and clinical condition. The LEPs were preliminarily identified on the original MICROMED apparatus. The examiner deleted the activities exceeding 65 μ and the ocular artifacts from the average using an automatic artifact rejection included in the MICROMED Brain Quick analysis software^2^. The analysis proceeded with the visual individuation of the main LEP components in all patients: the N1 on the T3 or T4 channel, depending on the stimulation site, referred to as *Fz*, and the N2 and P2 over the vertex (Cz channel) (Valeriani et al., 1996). These derivations were used for latency and preliminary amplitude evaluations. Considering the importance of habituation in migraine pathophysiology, we also evaluated the habituation pattern. For its computation, the sequence of potentials was divided into three blocks, and we considered the average of at least three artifact-free consecutive potentials for each block (de Tommaso et al., 2017, 2005c). Habituation was computed for the N2-P2 complex given that N1 is small in amplitude and recognizable only if a consistent number of potentials is averaged. The ratio between the third and first blocks of averaged LEP potentials was considered as the habituation index. Values under 1 indicated habituation (de Tommaso et al., 2017).

For topographic analysis, we considered only patients recorded with 61 scalp electrodes (10 patients in the sham and 10 patients in the real nVNS groups). For topographical representation of LEP components and statistical results in single groups, we used the same software and artifact correction as reported above. Pre-processing was performed in functions considering 1 s as post-stimulus and 100 ms of pre-stimulus time at a sampling rate of 256 Hz and bandpass filters 0.1–70 Hz. Next, we applied a notch filter at 50 Hz (L:48, H:52).

### Statistical Analysis

#### Laser-Evoked Potentials

We used the repeated-measures analysis of variance, considering the LEP latencies and amplitudes as well as N2-P2 habituation and visual analog scale values in the three conditions T0-T1-T2, with the group (active vs. sham) as factor. We also computed main contrasts between the different conditions and for the interaction condition x group.

For topographical representation of LEP statistical analysis, we used the above described EEGLAB MATLAB software. For statistical event-related potential (ERP) analysis, we considered the time interval of the P2 component (300–350 ms). The same software computed the one-way repeated-measures analysis of variance and the pairwise comparison between the T0-T1 and T0-T2 and T1-T2 conditions with Bonferroni correction. We plotted channel measures of the comparisons showing statistical significance.

#### Resting-State EEG

For the right and left forehead conditions, we took pairwise differences between T0 and T1 and T0 and T2 and T1 and T2 for all channels. We then compared the active nVNS group with the sham group on every channel and frequency band. Non-parametric permutation two-sample *t*-tests (1000 permutations) with correction for multiple testing was used. For every permutation, the minimum and maximum *t*-values were stored. The value at the 97.5th percentile of the null distribution was used as an upper threshold, and the value at the 2.5th percentile was used as a lower threshold. Any data point in the observed data was considered statistically significant if the *t*-value was at least as large as the upper threshold or at least as small as the lower threshold.

We applied the linear regression test to compare the percent rate of LEP change and the clinical outcome (responsive attack/total attacks).

## Results

### Patients

Among the 40 patients included in PRESTO, twenty-nine agreed to participate in the sub-study. The neurophysiological evaluation was thus comprised into the PRESTO protocol. Patients who were initially recruited, were recorded with 32 channels. Two patients were not included in the final evaluation due to artifact activity that could not be corrected, causing track elimination. The final evaluation included 14 patients in the active group and 13 patients in the sham group (**Table 1**). In this group of patients the total number of attacks with pain relief at 30, 60, and 120 min (secondary endpoint) was slightly and non-significantly higher in the active group compared to the sham group (*P* = 0.57) (**Table 1**).

**Table 1:** Demographic and clinical features of Migraine patients.

	N	Sex	Age (years)	Total migraine attacks in treatment period	Responsive attacks/total attacks	Not associated sympt./tot. attacks
nVNS	14	6 M – 8 F	36.07 ± 13.39	3.14 ± 1.35	0.42 ± 0.42	0.52 ± 0.4
Sham	13	4 M – 9 F	40.8 ± 11.35	3.3 ± 1.25	0.33 ± 0.35	0.38 ± 0.34
				ANOVA 0.1 n.s.	ANOVA 0.31 n.s.	ANOVA 0.87 n.s.

*Results about number of total migraine attacks, the ratio of the number of responsive attacks and the number of total attacks, and the number of attacks without associated symptoms (i.e., nausea, vomiting, photophobia, and phonophobia) after 120 min of nVNS or Sham stimulation are reported*.

### Laser-Evoked Potentials

#### Amplitudes

**FIGURE 2:**
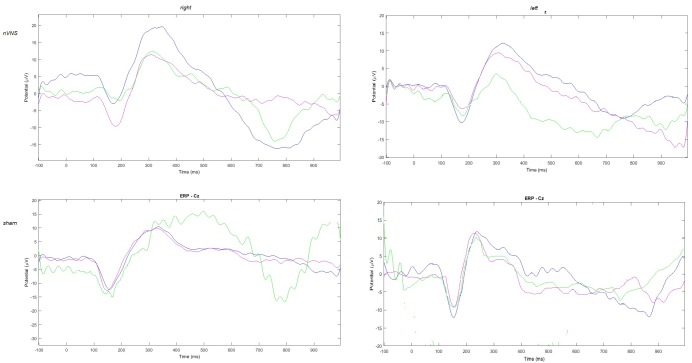
Grand average of laser-evoked potentials in the 14 patients submitted to single-session nVNS and 13 patients submitted to sham stimulation, plotted on the Cz channel. The three conditions are represented in different colors: T0 blue, T1 green, T2 pink.

The amplitude of the P2 component changed significantly for the effect of condition and group for the right side, and for the interaction between condition and group for the left side (**Table 2**). In the active group, the P2 evoked by the right and the left trigeminal branch was smaller during nVNS stimulation (T1) as compared to the T0 condition (**Table 2**). The sham device also attenuated the P2 amplitude evoked by the left trigeminal branch at T1 and T2, but this attenuation did not reach significance (**Figure 2**).

**Table 2:** Descriptive statistic of LEPs amplitudes, expressed in microvolts, obtained before, during and after nVNS and sham stimulation.

Left forehead		Mean N1	Erro r DS	95% CI		Mean N2	Error DS	95% CI		Mean P2	Error DS	95% CI	
				Lower	Upper			Lower	Upper			Lower	Upper
nVNS	T0	-3.15	1.27	-5.79	-0.52	-10	2.7	-15.63	-4.48	11.79	2.47	6.7	16.88
	T1	-2.41	0.87	-4.2	-0.62	-3.72	1.36	-6.53	-0.92	2.76	1.66	-0.674	6.2
	T2	-4.23	1.3	-6.92	-1.53	-9.88	2.27	-14.57	-5.19	7.56	1.79	3.87	11.26
Sham	T0	-5.22	1.32	-7.95	-2.49	-9.55	2.81	-15.33	-3.76	10.75	2.56	5.47	16.03
	T1	-3.35	0.9	-4.21	-1.49	-8.37	1.41	-11.29	-5.46	7.23	1.73	3.67	10.80
	T2	-2.30	1.35	-5.1	0.49	-7.61	2.36	-12.48	-2.74	5.35	1.86	1.51	9.18
ANOVA DF 2 error DF 24		*F*	*P*			*F*	*P*			*F*	*P*		
Condition		1.76	0.19			1.86	0.17			7.86	0.002		
Condition × group		1.31	0.28			2.12	0.14			3.78	0.37		
													
										Contrast : condition x group T0 vs. T1 *F* 6.03 *P* 0.021			

**Right forehead**		**Mean N1**	**Erro r DS**	**95% CI**		**Mean N2**	**Error DS**	**95% CI**		**Mean P2**	**Error DS**	**95% CI**	
				**Lower**	**Upper**			**Lower**	**Upper**			**Lower**	**Upper**

nVNS	T0	-3.810	1.34	-6.57	-1.04	-11.34	2.06	-15.61	-7.07	9.55	2.22	4.98	14.12
	T1	-3.64	0.67	-5.02	-2.26	-5.14	1.62	-8.49	-1.78	2.37	1.03	0.24	4.5
	T2	-2.91	1.33	-5.67	-0.163	-8.68	2.45	-13.75	-3.61	5.45	2.01	1.31	9.59
Sham	T0	-4.22	1.39	-7.09	-1.35	-6.41	2.06	-10.69	-2.14	5.84	2.3	1.10	10.59
	T1	-3.43	0.69	-5.86	0.99	-6.38	1.62	-9.73	-3.02	7.73	1.07	5.52	9.93
	T2	-3.6	1.38	-6.46	-0.750	-8.18	2.45	-13.25	-3.11	7.46	2.08	3.16	11.75
ANOVA DF 2 error DF 24		*F*	*P*			*F*	*P*			*F*	*P*		
Condition		0.83	0.20			2.76	0.082			1.6	0.21		
Condition × group		1.96	0.86			2.62	0.09			4.43	0.023		
													
										Contrast : condition x group T0 vs. T1 *F* 9.23 *P* 0.006			

*The active group included 14 patients, the sham group included 13 patients*.

A mild reduction of N2 was observed after the active stimulation, but it did not reach statistical significance. No changes were observed with N1.

#### Latencies

We did not detect any changes in wave latency after active or sham stimulation (**Supplementary Table 1**).

#### Topographical Analysis

**FIGURE 3:**
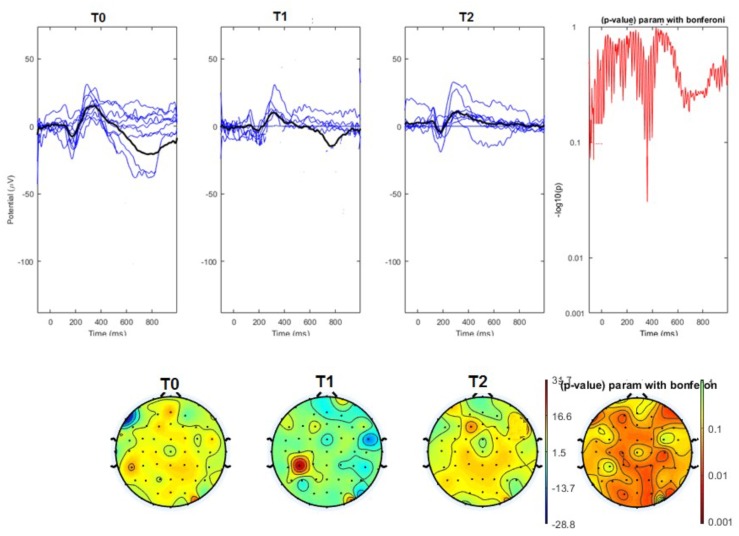
**(Upper)** Grand average and single laser-evoked responses from the right forehead, recorded on the Cz channel in the nVNS group. The values of the paired T0 vs. T1 student’s *t*-test corrected for multiple comparisons are reported on the right side. **(Bottom)** Two-dimensional scalp topography of the P2 wave in the three conditions and statistical probability map for the paired-comparison T0 vs. T1 conditions.

**FIGURE 4:**
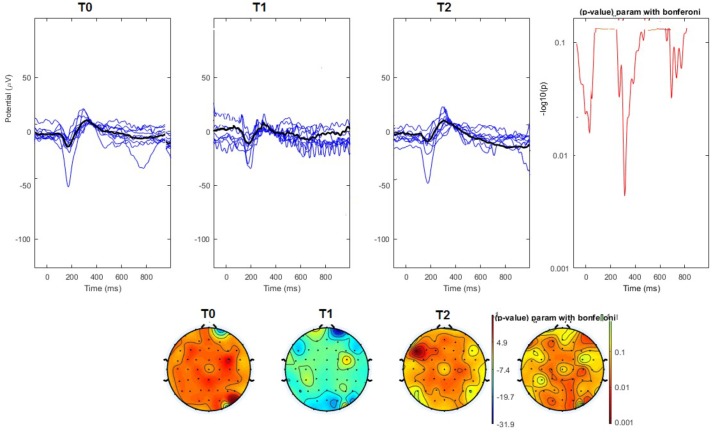
**(Upper)** Grand average and single laser-evoked responses from the left forehead, recorded the on the Cz channel in the nVNS group. The values of the paired T0 vs. T1 student’s *t*-test corrected for multiple comparisons are reported on the right side. **(Bottom)** Two-dimensional scalp topography of the P2 wave in the three conditions and statistical probability map for the paired-comparison T0 vs. T1 conditions.

**FIGURE 5:**
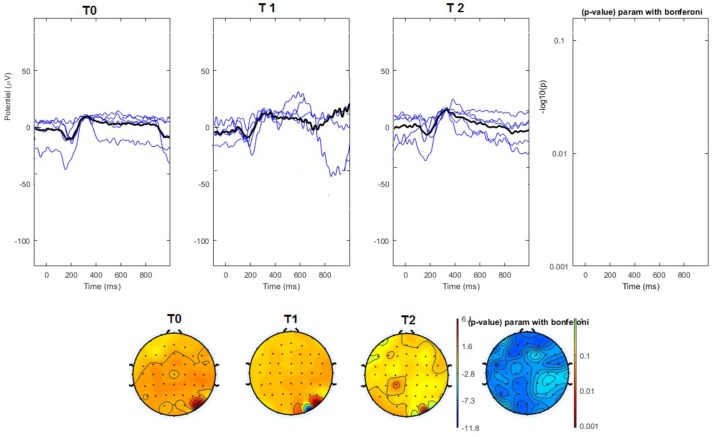
**(Upper)** Grand average and single laser-evoked responses from the right forehead, recorded the on the Cz channel in the sham group. The values of the paired T0 vs. T1 student’s *t*-test corrected for multiple comparisons are reported on the right side (no significant value). **(Bottom)** Two-dimensional scalp topography of the P2 wave in the three conditions and statistical probability map for the paired-comparison T0 vs. T1 conditions (no significant value).

**FIGURE 6:**
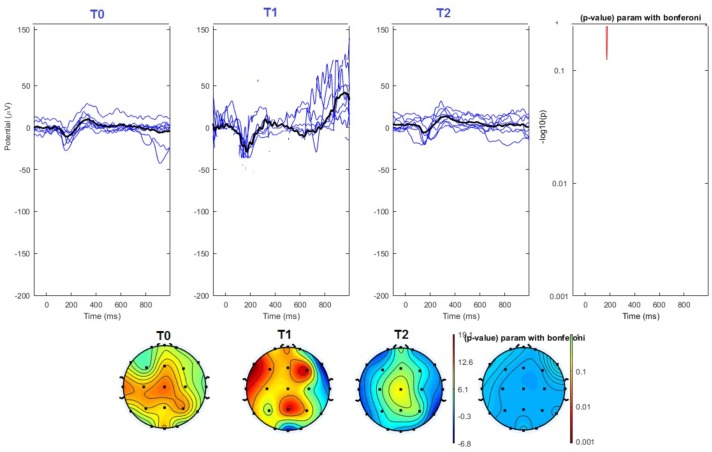
**(Upper)** Grand average and single laser-evoked responses from the left forehead, recorded the on the Cz channel in the sham group. The values of the paired T0 vs. T1 student’s *t*-test corrected for multiple comparisons are reported on the right side (no significant value). **(Bottom)** Two-dimensional scalp topography of the P2 wave in the three conditions and statistical probability map for the paired-comparison T0 vs. T1 conditions (no significant value). A reduced number of scalp derivations electrodes was used, and many bad channels were deleted.

The average values of plotted channels showed a reduction of the P2 wave during nVNS stimulation (T1) as compared with T0; the reduction was also present at T2 for the stimulation on the left side. In contrast with the sham stimulation (T1), we observed an increase of the P2 wave at both T0 and T2 (**Figure 2**). The statistical analysis on the 61 channels represented by 2-dimensional maps indicated a significant effect of nVNS in reducing the P2 component in the regions around the vertex (**Figures 3**, **4**). The comparison was significant when comparing T0 vs. T1. In the sham group, the presence of artifacts reduced the number of interpolated channels, especially on the left side, where an apparent increasing effect of sham stimulation was present on frontal, temporal, and centroparietal channels (**Figures 5**, **6**). However, no statistical significance emerged from the comparison of the different conditions in the migraine group treated with the sham device.

#### Habituation

The habituation index did not change at T1 and T2 under real and sham stimulation (**Table 3**).

**Table 3:** Descriptive statistic of subjective pain rating of laser stimuli, expressed as 0–100 VAS, and habituation index of N2P2 vertex complex and P2 wave.

Left forehead		Mean VAS	Err or DS	95% CI		Mean Hab N2P2	Err or DS	95% CI		Mean Hab P2	Err or DS	95% CI	
				Lower	Upper			Lower	Upper			Lower	Upper
nVNS	T0	55.21	6.01	42.81	67.61	0.86	0.14	0.56	1.16	1.11	0.56	0.7	2.1
	T1	50.85	6.10	38.27	63.43	0.79	0.44	-0.12	1.70	0.81	0.11	0.49	1.12
	T2	58.21	6.51	44.80	71.62	1.08	0.17	0.73	1.43	1	0.26	0.45	1.56
Sham	T0	61	6.24	48.13	73.86	1.07	0.15	0.75	1.38	0.98	0.23	0.81	1.42
	T1	59.76	6.33	46.71	72.82	0.68	0.46	-0.26	1.62	0.81	0.67	0.12	2.1
	T2	59.15	6.75	45.24	73.06	1.04	0.17	0.67	1.40	1.1	0.31	0.29	1.71
ANOVA DF 2 error DF 24		*F*	*P*			*F*	*P*			*F*	*P*		
Condition		0.70	0.49			0.52	0.59			0.81	0.43		
Condition × group		0.88	0.42			0.33	0.51			0.81	0.41		

**Right forehead**		**Mean VAS**	**Err or DS**	**95% CI**		**Mean Hab N2P2**	**Err or DS**	**95% CI**		**Mean Hab P2**	**Err or DS**	**95% CI**	
				**Lower**	**Upper**			**Lower**	**Upper**			**Lower**	**Upper**

nVNS	T0	66.64	5.45	55.40	77.87	0.66	0.1	0.44	0.88	0.99	0.11	0.60	1.41
	T1	62	5.18	51.32	72.67	0.75	0.32	0.09	1.42	0.6	0.23	0.01	1.31
	T2	66.14	5.55	54.70	77.58	1.05	0.19	0.66	1.45	1.1	0.12	0.52	1.52
sham	T0	53.38	5.66	41.72	65.04	0.80	0.11	0.57	1.03	1.1	0.19	0.9	1.71
	T1	61.30	5.38	50.22	72.39	0.90	0.33	0.22	1.59	1.32	0.31	0.75	2.3
	T2	58.92	5.76	47.05	70.79	0.85	0.19	0.44	1.26	1.21	0.21	0.69	1.71
ANOVA DF 2 error DF 24		*F*	*P*			*F*	*P*			*F*	*P*		
Condition		0.29	0.74			0.92	0.41			0.31	0.86		
Condition × group		2.17	0.13			0.51	0.6			0.07	0.37		

*Values below 1 indicated habituation. The active group included 14 patients, the sham group included 13 patients*.

#### Pain Rating

The laser pain did not change for the effect of active and sham devices (**Table 3**).

### Resting-State EEG

**FIGURE 7:**
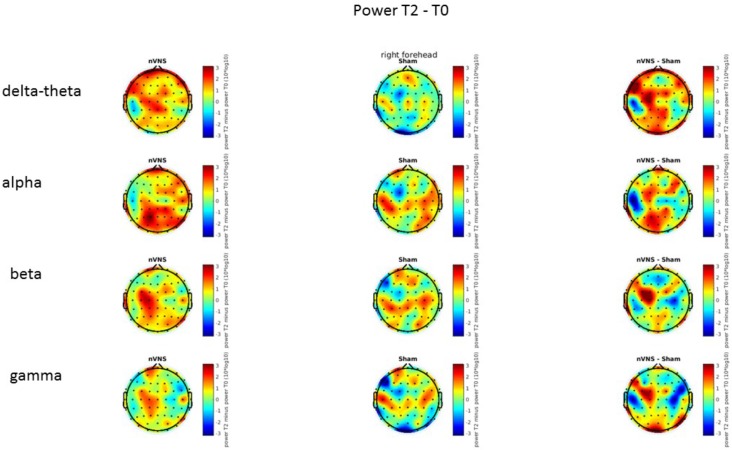
Difference (T2-T0) in the main EEG bands between the nVNS and sham groups for stimulation on the right side. The observed difference values are plotted in two-dimensional maps.

**FIGURE 8:**
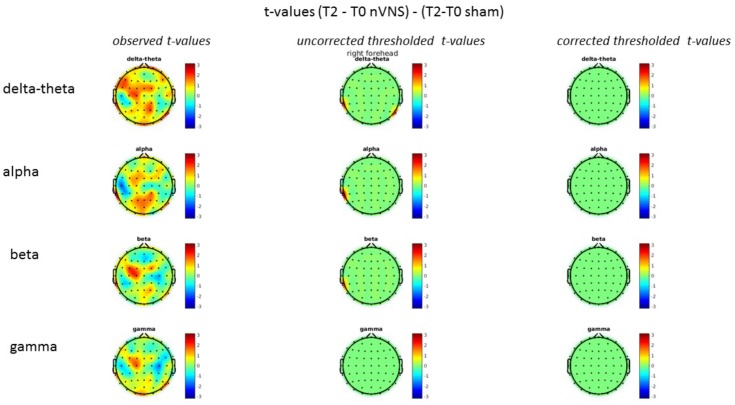
Comparison of the difference (T2-T0) in the main EEG bands between the nVNS and sham groups for stimulation on the right side. The observed and corrected *t*-values are plotted in two-dimensional maps.

**FIGURE 9:**
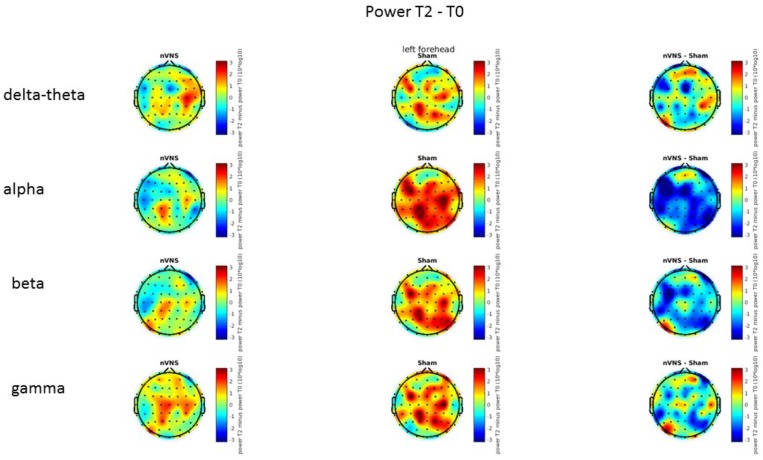
Difference (T2-T0) in the main EEG bands between the nVNS and sham groups for the stimulation of the left side. The observed difference values are plotted in two-dimensional maps.

**FIGURE 10:**
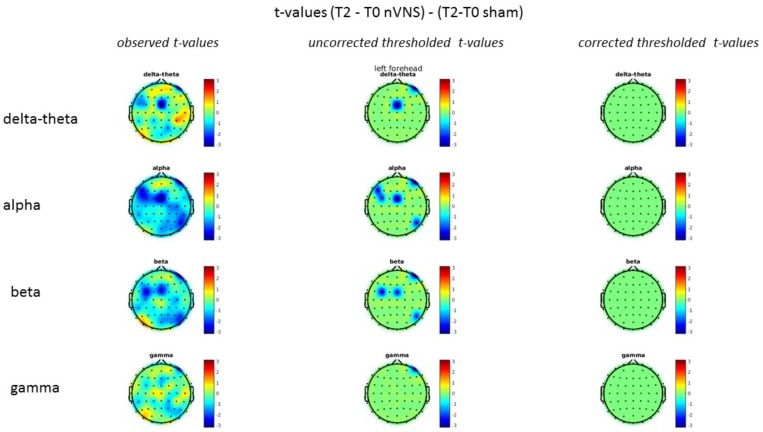
Comparison of the difference (T2-T0) in the main EEG bands between the nVNS and sham groups for stimulation of the left side. The observed and corrected *t*-values are plotted in two-dimensional maps.

**FIGURE 11:**
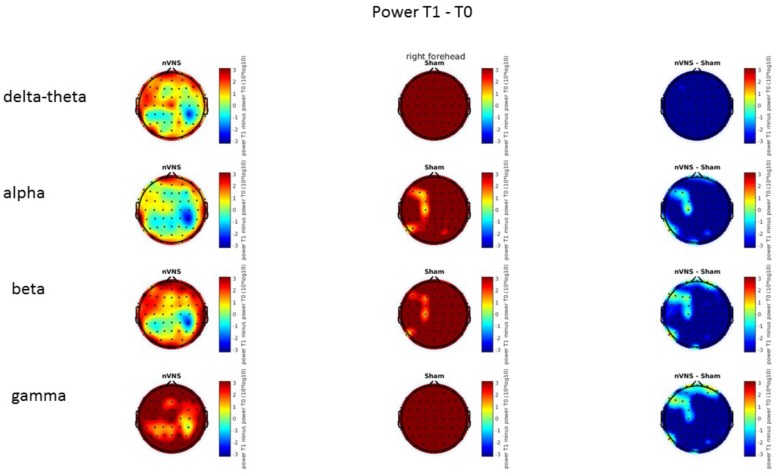
Difference (T1-T0) in the main EEG bands between the nVNS and sham groups for stimulation on the right side. The observed difference values are plotted in two-dimensional maps.

**FIGURE 12:**
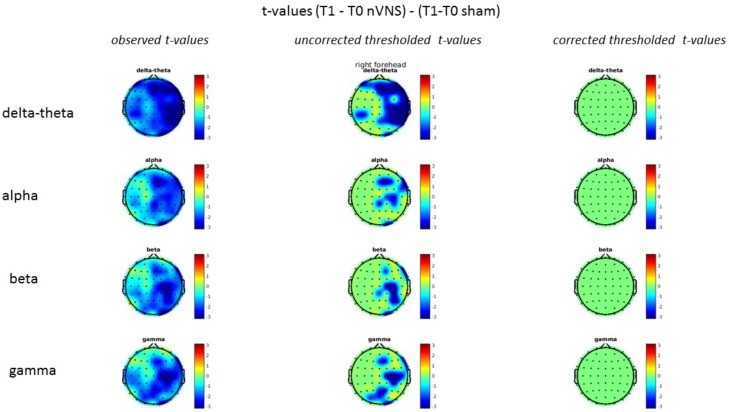
Comparison of the difference (T1-T0) in the main EEG bands between the nVNS and sham groups for stimulation on the right side. The observed and corrected *t*-values are plotted in two-dimensional maps.

**FIGURE 13:**
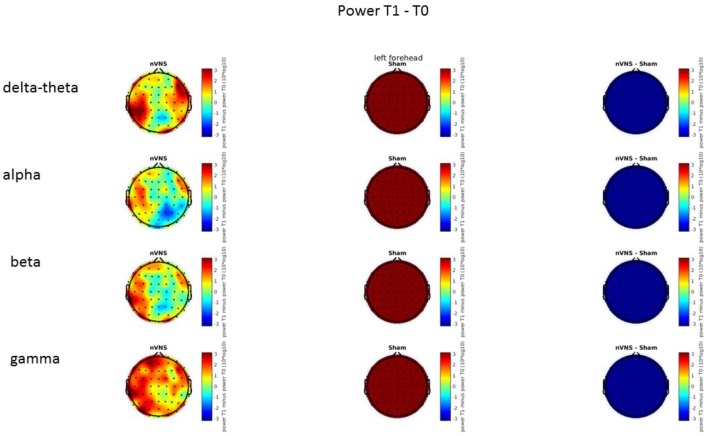
Difference (T1-T0) in the main EEG bands between the nVNS and sham groups for the stimulation of the left side. The observed difference values are plotted in two-dimensional maps.

**FIGURE 14:**
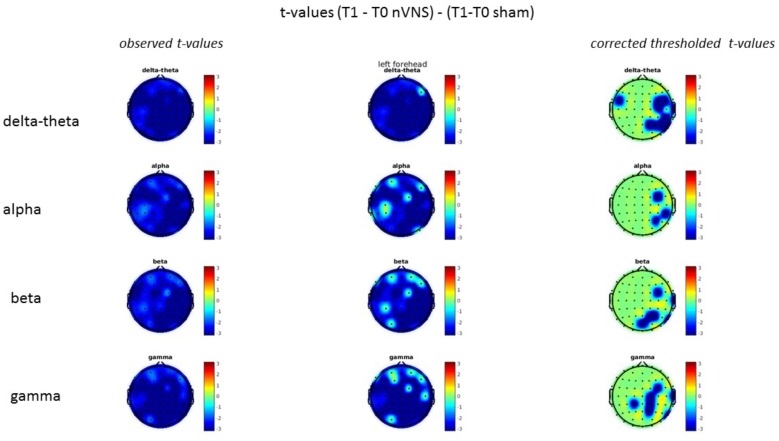
Comparison of the difference (T1-T0) in the main EEG bands between the nVNS and sham groups for stimulation of the left side. The observed and corrected *t*-values are plotted in two-dimensional maps.

On average, the EEG power increased at T2 compared to T0 in the nVNS group for all frequency bands. These changes were particularly evident in the right forehead condition. When comparing power increases (from T0 to T2) among the nVNS and sham groups, we observed that in the right forehead condition, the power increase was greater in the nVNS group than in the sham group, whereas in the left forehead condition, the power increase was lower in the nVNS group than in the sham group. However, when applying statistical tests with correction for multiple testing, neither of these between-group differences were found to be significant (**Figures 7**–**10**). The sham device (T1) caused an extreme increase in power for all considered frequency bands. For nVNS (T1), the power increase was largest in the gamma band but also present in the beta, alpha, and delta-theta frequency bands at the non-central electrodes. The higher power increase in the sham group compared to the nVNS group did not reach significance in the right-side stimulation condition. In the left-side stimulation condition, however, this higher power increase in the sham group compared to the nVNS group reached significance for isolated electrodes on the right side of the scalp at all frequency bands. These electrodes correspond mostly to regions where nVNS had no power increase effect, whereas sham had a power increase across the entire scalp (**Figures 11**–**14**).

#### Correlation With Clinical Outcome

**FIGURE 15:**
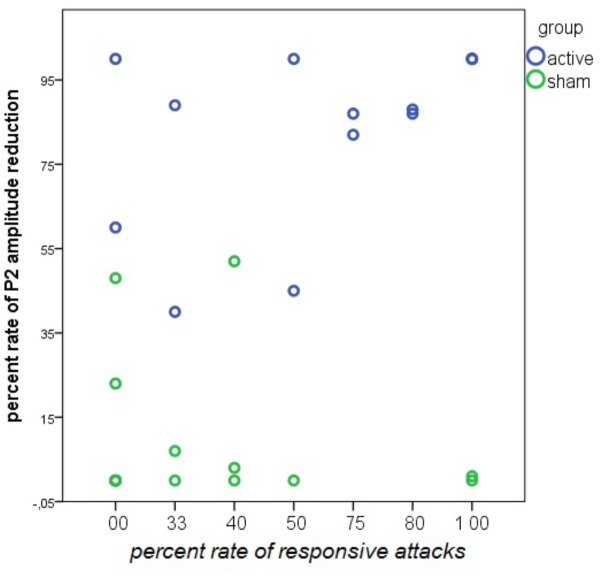
Linear regression analysis between the rate of responsive attacks and the rate of P2 wave reduction during nVNS and sham stimulation in 14 patients in the active and 13 patients in the sham group. The rate of P2 reduction was averaged across the left and the right forehead. In the nVNS group: β = 0.57; *t* = 2.44; *P* = 0.031. In the sham group: β = –0.24; *t* = –0.84; *P* = 0.41.

There was a positive relationship between the percentage of P2 reduction during nVNS and the percentage of responsive attacks. This relationship was absent in the sham group (**Figure 15**).

## Discussion

The active vagal nerve stimulation caused a reduction of cortical potentials evoked by ipsilateral trigeminal nociceptive stimulation, limited to the late positive component, without modifying the subjective pain perception or habituation of cortical potentials. This effect partially reverted shortly after vagal stimulation. It was correlated with the reduction of attack induced by nVNS. Resting-state EEG showed modest changes after right and left nVNS that were not significant compared to sham stimulation. The sham device seemed to cause an important perturbation of EEG rhythms, indicating some effects on brain activities, which needs to be distinguished from a pure artifact effect.

### Effect of Non-invasive Vagus Nerve Stimulation on Late Laser-Evoked Potentials

The absence of effects of nVNS on the earliest waves, especially on N1, appears to exclude an action of this stimulation modality on peripheral nerve endings and/or a direct modulatory effect on trigeminal spinal nucleus activity.

Animal studies demonstrated that the inhibitory effect on trigeminal nerves involves a central action on descending opioid and serotoninergic systems (Takeda et al., 1998; Tanimoto et al., 2002). Recently, Frangos and Komisaruk (2017) used fMRI to map brain regions that responded to cervical nVNS in healthy humans. nVNS activated the bilateral primary sensory cortex (S1), operculum, anterior-mid and posterior insula, dorsolateral prefrontal cortex, anterior cingulate cortex, supramarginal gyrus, thalamus, caudate, putamen, ipsilateral superior frontal and orbitofrontal cortices, supplementary motor area, contralateral occipital and middle temporal cortices, and cerebellum. Extensive deactivation was found bilaterally in visual areas the right hippocampus and parahippocampus, and the spinal trigeminal nuclei.

The present results do not support the hypothesis of a direct inhibition of the spinal trigeminal nuclei, which would have caused a total reduction of LEPs together with a delay of latencies. Our findings suggest a central action on the specific cortical areas generating the late vertex complex and especially the P2 wave. This effect was obtained with a not nociceptive stimulation, as the conventional cervical nVNS protocols cannot activate a-delta or C-fibers (Mourdoukoutas et al., 2018). These fibers input could thus interfere with the a-delta afferent volley elicited by laser stimulation, by inhibiting late responses at cortical level.

In our previous studies, we observed that over the course of a migraine attack, the P2 wave is amplified (de Tommaso et al., 2002, 2004), probably due to the activation of its cortical generator which we localized in the anterior cingulate cortex (de Tommaso et al., 2002), according to the pivotal studies on LEP dipolar modeling (Valeriani et al., 1996). Consistent with this interpretation, we have reported that acute pharmacological and non-pharmacological treatments for migraine, such as triptans and transcutaneous stimulation (de Tommaso et al., 2005c; Vecchio et al., 2017), are effective in reducing this wave and the bold signal from the anterior cingulate (Russo et al., 2017). Considering that fMRI studies showed nVNS activation of the anterior cingulate, this type of stimulation interferes with the cortical reception of painful stimuli, with a first come, first served mechanism (Garcia-Larrea, 2004). Recent fMRI studies of migraine patients indicated a basal dysfunction of the so called *salience* network, in which the anterior cingulate has a primary position (Androulakis et al., 2017). This basal dysfunction of the cortical areas elaborating relevant stimuli with special regard to pain may facilitate migraine attack persistence. The concomitant activation of part of this network produced by nVNS may thus reduce headache persistence and interrupt the course of a migraine attack (Tassorelli et al., 2018). Other cortical areas outside the LEP generators are activated by nVNS [6] and may contribute to the resolution of a migraine attack.

The inhibitory effect of nVNS on late LEPs was short-term, as the P2 wave partially recovered soon after the end of stimulation. A similar temporary inhibition was also reported after transcutaneous electrical stimulation (Vecchio et al., 2017). This short-term effect may be a limit for the efficacy of these devices. Further studies are needed to clarify whether the repetition of stimulation may prolong the neurophysiological effects, as seen in a number of animal models of nVNS effects (Simon and Blake, 2017).

The sham device created a significant interference with EEG recordings, with interposed artifactual activities and a probable real influence on EEG rhythms (see below). The sham electrical stimulation was clearly perceived by the patients, thus creating an attentive competition against the laser stimuli. In some patients, it reduced vertex waves, which generate from cortical areas within the salience network and are strongly influenced by attention deviation (Iannetti et al., 2008). The direct central action of nVNS on cortical areas generating late LEPs could explain the net effect of active stimulation, subtracting the sham effect linked to cognitive factors.

Migraine patients have a high level of attention toward painful stimuli, especially those delivered at the trigeminal level, and are hardly distracted from these (de Tommaso et al., 2007, 2008). The pure cognitive action with distracting procedures could be ineffective in modulating the trigeminal nociceptive system.

Non-invasive vagus nerve stimulation was not effective in resolving the reduced habituation of the LEP vertex complex and P2 wave in the migraine sample, though we observed a slight recovery of this pattern. The migraine patients included in the present study confirmed the lack of vertex LEP amplitude decline during stimulation, as compared with normative data from our laboratory (de Tommaso et al., 2017). Complex phenomena of cognitive origin and the altered excitability of the nociceptive system influence LEP-reduced habituation in migraine. In contrast with other types of sensory modality, the LEP-habituation deficit persists during the entire migraine cycle including the critical phase. Therapeutic interventions that are able to induce a long-term modulation of the nociceptive trigeminal system resolved this habituation deficit (de Tommaso et al., 2005a, 2016; Vecchio et al., 2016). Single sessions of transcutaneous stimulation in the trigeminal territory provided an uncertain effect on LEP habituation in migraine patients, though it reduced the vertex wave amplitude (Vecchio et al., 2017). The short-term effect of nVNS on cortical areas generating late LEPs may not be sufficient to resolve the pattern of reduced habituation to trigeminal noxious stimuli.

Non-invasive vagus nerve stimulation did not modify subjective pain ratings of laser stimuli, consistent with other studies exploring the effects of single sessions of cortical or transcutaneous stimulation on trigeminal pain-related cortical responses (de Tommaso et al., 2010; Vecchio et al., 2016, 2017). This finding was expected as it is in agreement with the largely accepted hypothesis that LEPs are not the neurophysiological signature of subjective pain ratings, while they rather express the functional status of pain pathways (Mouraux and Iannetti, 2009).

### Resting-State EEG

Electroencephalogram studies of VNS effects in epileptic patients showed clear effects on theta rhythm synchronization and an increase of gamma power (Marrosu et al., 2005). A recent study in Crohn’s disease patients who were implanted with a low-frequency left vagal nerve stimulation showed an acute effect with delta and theta spectral power increases and a 12-month chronic reducing effect on alpha activity (Kibleur et al., 2018). In our migraine sample, the peripheral stimulation of the right and left vagal nerve at a frequency of 25 Hz induced an increase in the slow delta-theta and beta-gamma power. A proper interpretation of these findings in our study is not possible due to our sham stimulation generating a general increase in all frequency bands. This suggests that the sham device generated extra cerebral activity of muscular or pure electrical origin, although we spent time in visual and automatic detection methods to attempt to delete and correct the artifacts. We cannot presently rule out that a single nVNS session might have induced a modulation of slow and fast EEG rhythms. The potential contribution of basal EEG modulation to improve or resolve a single migraine attack is a matter of debate. A possible interference with the CSD phenomena seems quite speculative because the occurrence of CSD in migraine attacks without aura is debatable (Ayata, 2010; Borgdorff, 2018). Alternatively, the possible acute modulation of EEG rhythms may be a sign of the central action of nVNS on a large range of associative cortical areas involved in attentional behavior and descending pain control.

The small number of cases in our sub-study does not allow a definite conclusion about the possible clinical significance of present results, and in this sub-study, the number of responsive attacks was not significantly higher in the nVNS arm than in the placebo group. However, the reduction of the P2 wave was evident in the nVNS responders but absent in patients with a clinical response to the sham device. This supports an nVNS effect on the cortical areas modulating trigeminal pain, not only in experimental conditions, but also during trigeminal activation over the course of a migraine attack. Present results could confirm that LEPs are a reliable method to test the mechanism of action of drugs and non-pharmacological interventions in migraine attacks.

### Study Limitations

This study was exploratory in nature and admittedly conducted on a low number of patients who agreed with the neurophysiological evaluation at the time of randomization for the PRESTO trial. This did not allow for robust correlations with clinical outcomes.

The second limitation is represented by the physical feature of the sham device, which was designed to induce a cognitive distraction via a clearly perceived sensation, without considering a possible interference with the brain EEG rhythms or even a potential low level of vagal stimulation. Sham stimulation caused a heavy artifact interference with EEG activity, which required a complex procedure of EEG cleaning and correction to obtain reliable event-related responses.

Lastly, the EEG recording was short, according to the nVNS and sham stimulation duration. The resting state EEG was partly extracted from the laser inter-stimuli intervals, which may be questionable. However, we decided to maintain nVNS and sham duration as similar as possible to that used for therapeutic purposes.

## Conclusion

The findings obtained in the present EEG and LEP study in a subgroup of patients enrolled in the PRESTO trial suggest that nVNS acts on the cortical areas that are responsible for trigeminal pain control. These findings also pave the ground for future studies aimed at confirming possible correlations with clinical outcomes, including the effect on symptoms directly correlated with trigeminal pain processing and modulation, such as headache intensity, pain extension, and allodynia.

## Data Availability

The datasets generated for the PRESTO study can be found at ClinicalTrials.gov [https://www.clinicaltrials.gov/ct2/show/NCT02686034].

## Author Contributions

EV contributed to study design, clinical assessment, data analysis, and manuscript preparation and editing. IB contributed to data and statistical analysis. KR contributed to neurophysiological assessment and data acquisition. CT and EL contributed to manuscript editing. MdT contributed to conception and study design, clinical assessment, statistical analysis, and manuscript preparation and editing.

## Conflict of Interest Statement

CT has received consultancy fees from Allergan S.p.A., electroCore, LLC, Eli Lilly and Company, and Novartis AG and research grants from the European Commission and the Italian Ministry of Health. She is also a principal investigator or collaborator for RCTs sponsored by Alder BioPharmaceuticals Inc., Eli Lilly and Company, and Teva Pharmaceutical Industries Ltd. EL is an employee of electroCore, LLC, and receives stock ownership. MdT has received advisory fees from Allergan S.p.A., Neopharmed, and Pfizer Inc. The remaining authors declare that the research was conducted in the absence of any commercial or financial relationships that could be construed as a potential conflict of interest.

## Footnotes

1 https://sccn.ucsd.edu/wiki/Makoto’s_preprocessing_pipeline

2 http://www.micromed.eu/en-us/products/ID/1/BRAIN-QUICK--EEG-Line

## Abbreviations

CSD: cortical spreading depression
EEG: electroencephalogram
fMRI: functional magnetic resonance imaging
LEP: laser-evoked potential
nVNS: non-invasive vagus nerve stimulation
PRESTO: PRospectivE Study of non-invasive vagus nerve stimulation for the acute Treatment Of migraine
VNS: vagus nerve stimulation
