# Supplementary material for: Effect of Non-invasive Vagus Nerve Stimulation on Resting-State Electroencephalography and Laser-Evoked Potentials in Migraine Patients: Mechanistic Insights
Source: Front Hum Neurosci. 2018 Sep 13;12:366. doi: 10.3389/fnhum.2018.00366 (PMC6146235; doi:10.3389/fnhum.2018.00366)
Supplement: Supplementary file 1 [file Table_1.DOCX]

| Left forehead condition | | Mean | Error DS | 95% CI | | Mean | Error DS | 95% CI | | Mean | Error DS | 95% CI |  |
| --- | --- | --- | --- | --- | --- | --- | --- | --- | --- | --- | --- | --- | --- |
|  |  |  |  | Lower | Upper |  |  | Lower | Upper |  |  | Lower | Upper |
| nVNS | T0 | 136.172 | 2.476 | 130.722 | 141.622 | 198.672 | 4.495 | 188.961 | 208.383 | 314.757 | 6.000 | 301.888 | 327.626 |
|  | T1 | 129.785 | 4.016 | 120.946 | 138.625 | 185.742 | 6.326 | 172.075 | 199.410 | 311.319 | 8.479 | 293.135 | 329.504 |
|  | T2 | 136.133 | 2.485 | 130.664 | 141.602 | 187.930 | 5.702 | 175.612 | 200.248 | 301.771 | 10.667 | 278.892 | 324.650 |
| sham | T0 | 130.156 | 3.132 | 123.263 | 137.049 | 195.045 | 4.805 | 184.663 | 205.426 | 317.299 | 6.803 | 302.707 | 331.891 |
|  | T1 | 126.156 | 5.08 | 114.975 | 137.337 | 185.938 | 6.763 | 171.326 | 200.549 | 319.576 | 9.614 | 298.956 | 340.195 |
|  | T2 | 133.969 | 3.143 | 127.051 | 140.886 | 184.018 | 6.095 | 170.850 | 197.186 | 303.393 | 12.096 | 277.450 | 329.335 |
| ANOVA DF 2 | | F | P |  |  | F | p |  |  | F | p |  |  |
| condition |  | 3.58 | 0.067 |  |  | 2.12 | 0.14 |  |  | 1.43 | 0.27 |  |  |
| condition x group | | 0.4 | 0.67 |  |  | 0.74 | 0.33 |  |  | 2.10 | 1.15 |  |  |
| Rigth forehead condition | | Mean | Error DS | 95% CI |  | Mean | Error DS | 95% CI |  | Mean | Error DS | 95% CI |  |
|  |  |  |  | Lower | Upper |  |  | Lower | Upper |  |  | Lower | Upper |
| nVNS | T0 | 139.941 | 2.533 | 134.297 | 145.585 | 186.250 | 4.081 | 177.434 | 195.066 | 322.149 | 5.996 | 309.195 | 335.102 |
|  | T1 | 131.309 | 3.148 | 124.293 | 138.324 | 181.719 | 7.442 | 165.641 | 197.797 | 316.641 | 9.959 | 295.125 | 338.156 |
|  | T2 | 137.656 | 2.106 | 132.963 | 142.350 | 182.207 | 7.306 | 166.423 | 197.991 | 303.457 | 12.446 | 276.568 | 330.346 |
| sham | T0 | 129.532 | 3.582 | 121.550 | 137.513 | 177.098 | 4.363 | 157.673 | 176.523 | 326.630 | 6.410 | 312.782 | 340.477 |
|  | T1 | 122.695 | 4.452 | 112.774 | 132.616 | 181.942 | 7.956 | 164.754 | 199.130 | 323.281 | 10.647 | 300.280 | 346.282 |
|  | T2 | 133.438 | 2.979 | 126.800 | 140.075 | 178.505 | 7.810 | 161.631 | 195.378 | 307.098 | 13.306 | 278.353 | 335.843 |
| ANOVA DF 2 | | F | p |  |  | F | p |  |  | F | p |  |  |
| condition |  | 4.16 | 0.052 |  |  | 0.63 | 0.54 |  |  | 2.42 | 0.1 |  |  |
| condition x group | | 0.54 | 0.59 |  |  | 2.13 | 0.16 |  |  | 0.014 | 0.98 |  |  |

Table 1 S Descriptive statistic of LEPs latencies, expressed in msec, obtained by left and right forehead stimulation before, during and after nVNS and sham device. The active group included 14 patients, the sham group included 13 patients.
